# Influence of diurnal phase on behavioral tests of sensorimotor performance, anxiety, learning and memory in mice

**DOI:** 10.1038/s41598-021-03155-5

**Published:** 2022-01-10

**Authors:** Chi-Hui Tsao, Jonathan Flint, Guo-Jen Huang

**Affiliations:** 1 Department and Graduate Institute of Biomedical Sciences, College of Medicine, Chang Gung University, Taoyuan, 33302 Taiwan; 2 Center for Neurobehavioral Genetics, Semel Institute for Neuroscience and Human Behavior, University of California, Los Angeles, Los Angeles, CA 90095 USA; 3 Department of Neurology, Chang Gung Memorial Hospital-Linkou Medical Center, Taoyuan 333, Taiwan; 4 Healthy Aging Research Center, Chang Gung University, Taoyuan, 33302 Taiwan; 5 Molecular Medicine Research Center, Chang Gung University, Taoyuan, 33302 Taiwan

**Keywords:** Circadian rhythms and sleep, Emotion, Learning and memory, Behavioural methods

## Abstract

Behavioral measurements in mice are critical tools used to evaluate the effects of interventions. Whilst mice are nocturnal animals, many studies conduct behavioral tests during the day. To better understand the effects of diurnal rhythm on mouse behaviors, we compared the results from behavioral tests conducted in the active and inactive phases. C57BL/6 mice were used in this study; we focus on sensorimotor performance, anxiety, learning and memory. Overall, our results show mice exhibit slightly higher cutaneous sensitivity, better long-term contextual memory, and a greater active avoidance escape response during the active phase. We did not observe significant differences in motor coordination, anxiety, or spatial learning and memory. Furthermore, apart from the elevated-O-maze, there was no remarkable sex effect among these tests. This study provides information on the effects of different diurnal phases on types of behavior and demonstrates the importance of the circadian cycle on learning and memory. Although we did not detect differences in anxiety and spatial learning/memory, diurnal rhythm may interact with other factors to influence these behaviors.

## Introduction

Mouse behavioral testing is an important methodology for assessing the impact of pharmaceutical interventions and genetic engineering. Mouse models are widely used for scientific investigation and are particularly prevalent in neuroscience. Among all the mouse strains, C57BL/6 is one of the most popular with neuroscientists to study brain functions. From previous high-throughput phenotyping data, C57BL/6 has been shown to be less anxious compared to other inbred strain of mice in behavioral tests. This feature makes it a more suitable strain for behavioral assays^1^.

However, mice are nocturnal animals; and their active phase is during the night. Given that most scientists work during daytime, many studies are conducted during the natural sleep phase of mice. There are two issues here. First, sleep disruption can alter emotional responses and cognition^2,3^. Second, many physiological processes including hormone secretions, such as corticosterone, and gene expression, which influence behavioral performance, are regulated by the circadian clock^4,5^. However, it is uncommon for research institutes to maintain mice under a reverse light system in animal houses. Thus, many behavioral assays are conducted during their inactive phase when mice are normally asleep. If mice perform quite differently during the active phase compared to the inactive phase, then this may partially explain some contradictory results between different published studies. Numerous studies have demonstrated the importance of circadian clocks on mood disorders and learning^6,7^. So far, there are a limited number of published documents investigating the anxiety and learning performance at different times of the day^8–10^. In this study, we aimed to investigate whether mice performed differently in behavioral tests during their active and inactive phases. Specifically, we focused on hippocampal-related anxiety and learning memory, which have been studied extensively. The behaviors we assayed include motor coordination, cutaneous sensitivity, anxiety, learning and memory. Despite some slight differences detected in a few tests, overall our results demonstrate that there are no dramatic discrepancies in behavioral performance conducted during active and inactive phases.

## Results

**Figure 1:**
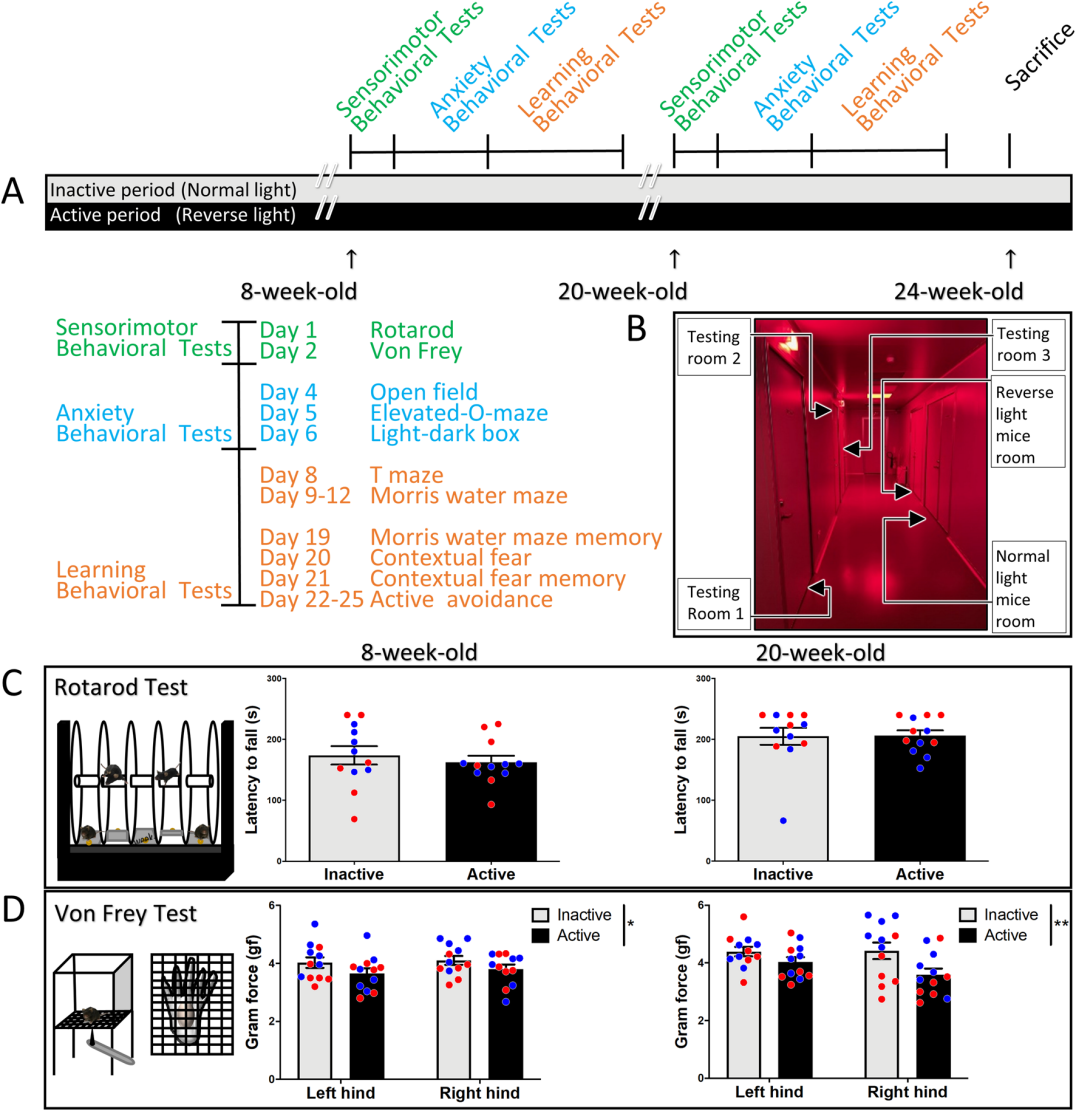
Effects of diurnal phase on motor coordination and sensory stimulation. (**A**) Time course of the experimental procedure. Two groups of mice (n = 12; 6 males and 6 females in each group) were housed in normal light and reverse light condition. Behavioral tests were conduct 2 h after lights on/off (ZT 2 for inactive group, and ZT 14 for active group). (**B**) The relative position of animal rooms (one reverse light, one normal light) and behavioral testing rooms of the animal house unit. (**C**) Illustration of rotarod test (left), there is no difference detected in the latency to fall between active group and inactive group in neither the initial test (middle), nor the retest 3 months later (right). (**D**) For von Frey test, mice tested in the active phase exhibit a slightly more sensitive in cutaneous sensory testing than mice tested in the inactive phase in the initial tests. Three months later, mice tested in the active phase still exhibit more sensitivity to filament stimulus compared to mice tested in inactive phase. Blue circles: male mice; Red circles: female mice.

To understand the effects of circadian phase on mice behavior, 24 mice were divided into two groups when 4 week-old (active phase: mice were maintained in reverse light; inactive phase: mice were maintained in normal light). All mice (8 week-old) during the active and inactive phases were subjected to a battery of behavioral tests (initial test), including: rotarod, von Frey test, open field, elevated-O-maze, light–dark box, water maze, T-maze, contextual fear conditioning, and active avoidance. To further confirm the results at a different age, all the behaviors were repeated again three months later (retest, 20 week-old) (Fig. 1A). Behavioral testing was started two hours after lights on/off; Zeitgeber Time (ZT) 14 for the active group and Zeitgeber Time (ZT) 2 for the inactive group. Ten minutes before the test, tested cages were relocated from animal room to the waiting hallway (red dim light, 15 lx) to minimize the effect of light (Fig. 1B). Tests were then conducted under the following light intensities: open field (400 lx), elevated-O-maze (350 lx), light–dark box (440 lx), water maze (440 lx), T-maze (400 lx), contextual fear conditioning (inside the dark box), and active avoidance (inside the dark box).

### Effects of diurnal phase on motor coordination and sensory stimulation

To test the influence of the light–dark cycle on motor coordination and sensory stimuli, mice were subjected to the rotarod and von Frey tests. Our results show that there are no differences on the latency to fall in the rotarod test between inactive and active groups in the initial test (*p* = 0.54, *t*_(22)_ = 0.61), nor the retest three months later (*p* = 0.94, *t*_(22)_ = 0.07) (Fig. 1C). Next, to assess sensitivity to sensory stimuli, all mice were subjected to the von Frey test; a mechanical stimulation by a filament to hind paws. Mice tested in the inactive phase are slightly less sensitive in the initial test (*p* = 0.049, *F*_(1,44)_ = 4.06). For the retest three months later, the results are similar to the initial test, mice tested in inactive phase are still less sensitive than tested in active phase (*p* = 0.0085, *F*_(1,44)_ = 7.59) (Fig. 1D). These results suggest that the performance of motor coordination and balance is not influenced by diurnal activity, but cutaneous sensitivity is more responsive in the active phase.

### Effects of diurnal phase on anxiety tests

**Figure 2:**
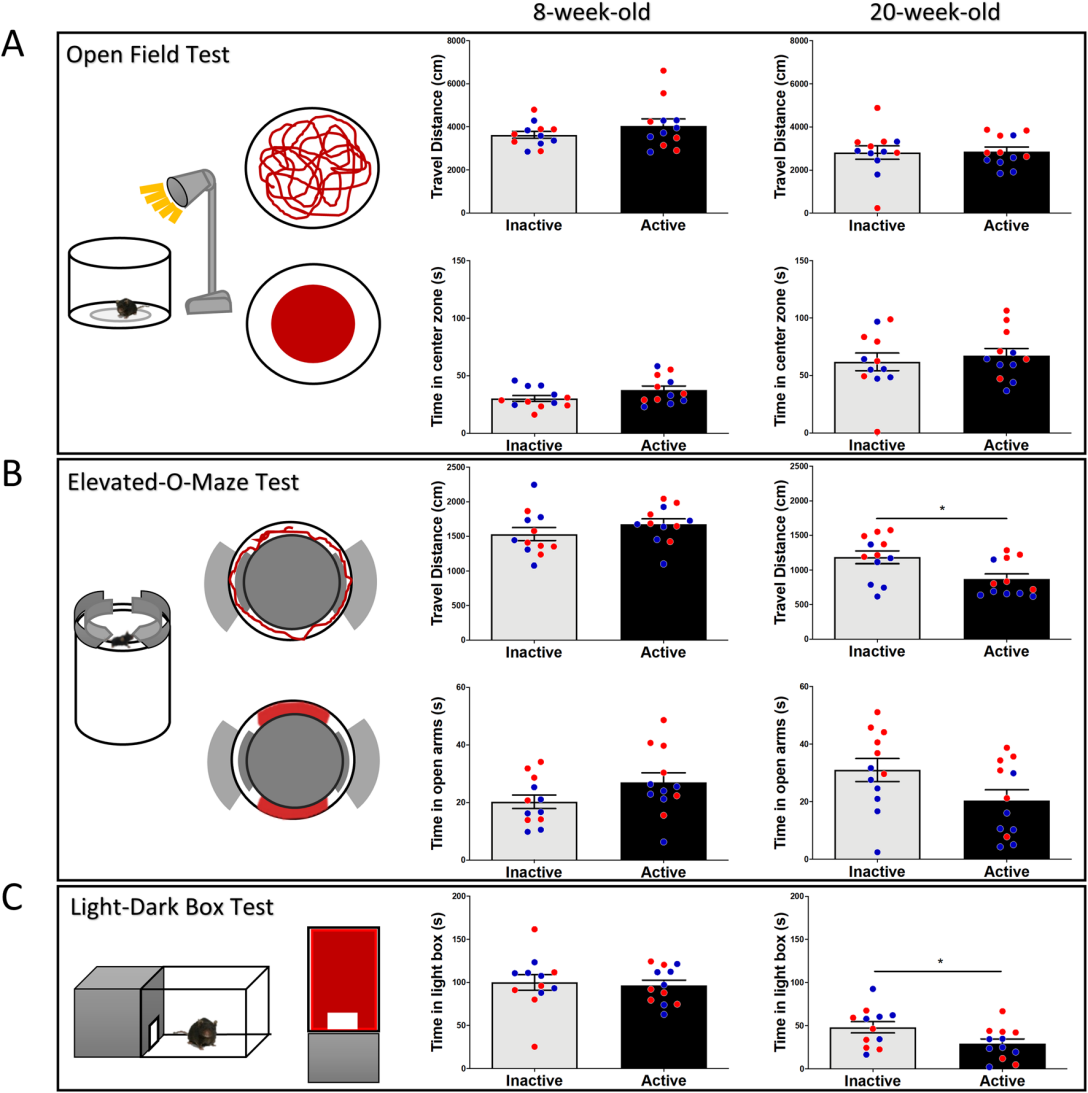
Effects of diurnal phase on anxiety tests. (**A**) There is no significant difference in distance traveled or time spent in the center zone of open field between active and inactive groups in the initial test, nor in the retest 3 months later. (**B**) In the elevated-O-maze, there is no significant difference detected in distance travel or times spent in the open arms between active and inactive groups. However, 3 months later, the mice from the inactive group travelled a higher distance, but with no significant difference in time spent in the open arms in the retest. (**C**) In the light–dark box, there is no significant difference in time spent in the light box in the initial test. In the retest 3 months later, mice tested in active phase spent less time in the light compartment. Blue circles: male mice; Red circles: female mice.

To better understand the effects of diurnal rhythm on anxiety, we chose three common tests associated with anxiety like behavior: the open field test, elevated-O-maze, and light–dark box. All mice were subjected to these three tests. Our results show that there were no significant differences detected between inactive and active groups in the open field test in the initial test (travel distance, *p* = 0.25, *t*_(22)_ = 1.17; time in center zone, *p* = 0.09, *t*_(22)_ = 1.72), nor the retest three months later (travel distance, *p* = 0.89, *t*_(22)_ = 0.13; time in center zone, *p* = 0.57, *t*_(22)_ = 0.56) (Fig. 2A). For the elevated-O-maze test, there were no significant difference detected in the initial test (travel distance, *p* = 0.244, *t*_(22)_ = 1.19; during in the open arms, *p* = 0.11, *t*_(22)_ = 1.64). However, mice tested in inactive period showed higher travel distance (*p* = 0.01, *t*_(22)_ = 2.62) and exhibited a trend to stay longer in the open arms (*p* = 0.06, *t*_(22)_ = 1.94) in the retest (Fig. 2B). Next, we subjected all the mice to the light–dark box test, in the initial test there were no differences in time spent in the light compartment: *p* = 0.75, *t*_(22)_ = 0.31). Three months later, all mice were subjected to the tests again. Mice tested in the inactive period spent more time in the light compartment (*p* = 0.036, *t*_(22)_ = 2.23) (Fig. 2C). The results show that there were no remarkable differences observed in anxiety tests while testing mice during active or inactive periods in the first test. However, three months later upon retesting, we did observe that mice exhibited less travel distance in the elevated-O-maze and spent less time in the light compartment.

### Effects of diurnal phase on spatial learning and memory

**Figure 3:**
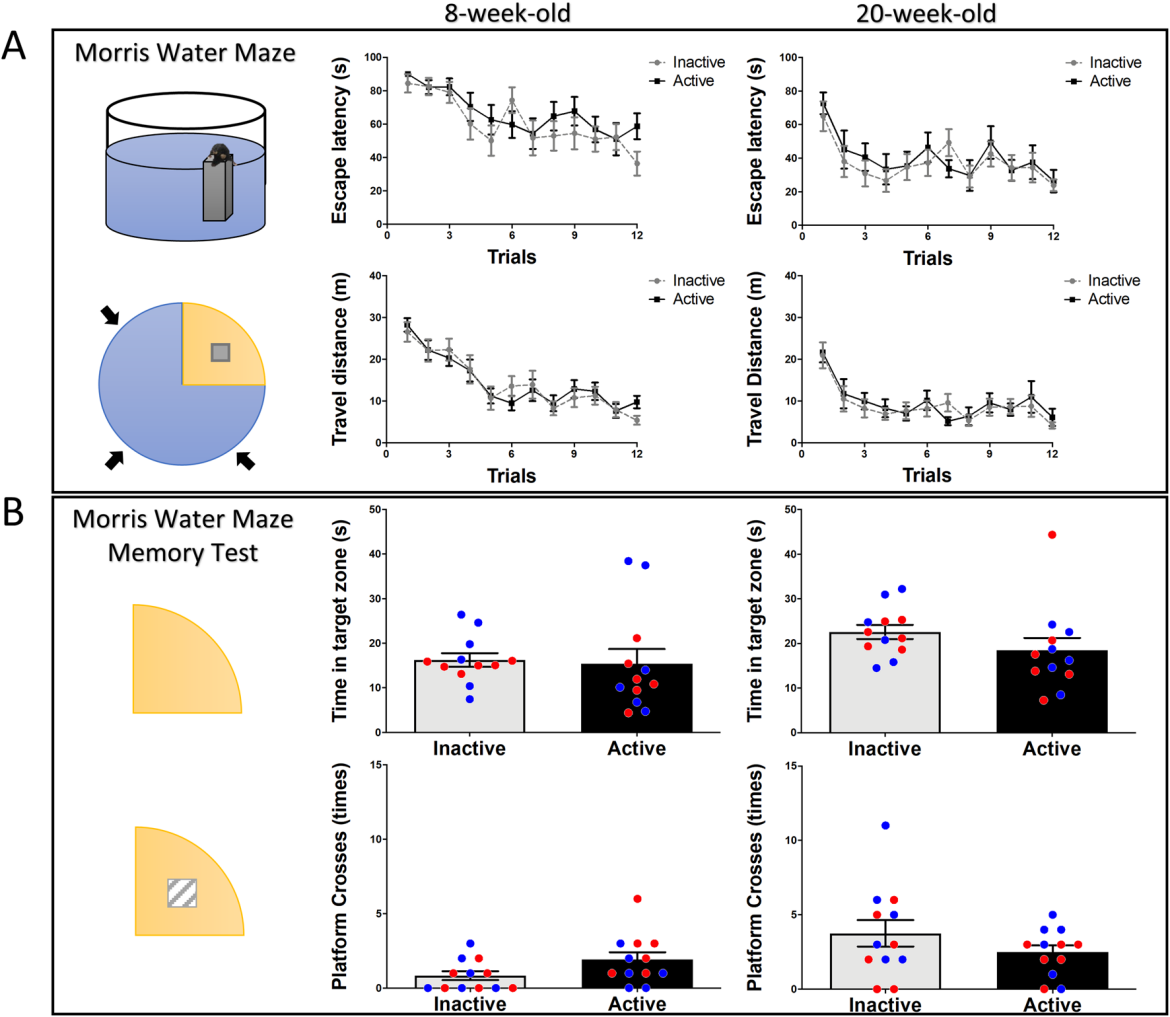
Effects of diurnal phase on spatial learning and memory. (**A**) During water maze training, there is no difference in escape latency or travel distance between active and inactive groups, neither in the retest 3 months later. (**B**) For the memory retrieval of the water maze, there is no significant difference detected in time in target zone or platform crosses 7 days after the last training, nor in the retest. Blue circles: male mice; Red circles: female mice.

It is unclear whether a time-of-day may influence the performance of spatial learning and memory. To investigate the effects of diurnal rhythm on spatial learning and memory, the water maze was used. All mice were subjected to the water maze for 4 days to examine acquisition. The results show there were no differences in escape latency (*p* = 0.24, *F*_(1,22)_ = 1.45) and travel distance (*p* = 0.85, *F*_(1,22)_ = 0.03) in the initial test, nor the retest three months later (escape latency, *p* = 0.53, *F*_(1,22)_ = 0.39; travel distance, *p* = 0.65, *F*_(1,22)_ = 0.2) (Fig. 3A). Seven days after the last training, the escape platform was removed, and mice were subjected to the water maze, and spatial memory was evaluated. There were no significant differences detected between the active group and inactive group for the initial test (time spent in target zone, *p* = 0.82, *t*_(22)_ = 0.22; platform crosses, *p* = 0.06, *t*_(22)_ = 1.9), nor the retest three months later (time spent in target zone, *p* = 0.21, *t*_(22)_ = 1.28; platform crosses, *p* = 0.22, *t*_(22)_ = 1.25) (Fig. 3B). These data suggest that there are no obvious differences detected in spatial learning and memory between mice tested in active or inactive period.

### Effects of diurnal phase on T-maze, contextual fear, and active avoidance, and the expression of clock genes

**Figure 4:**
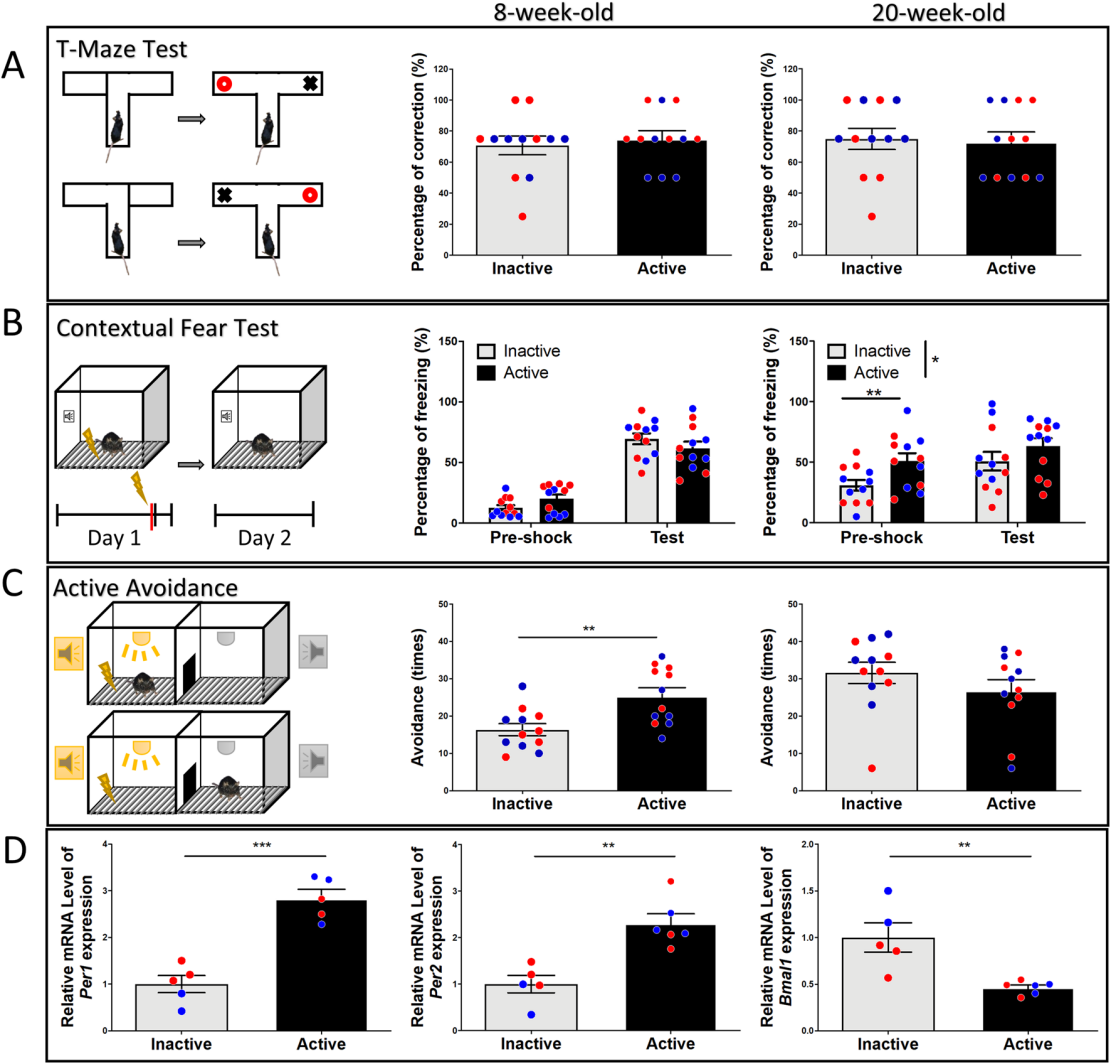
Effects of diurnal phase on T-maze, contextual fear, active avoidance, expression of clock genes. (**A**) In the T-maze test, there is no significant difference in the percentage of correct choices observed influenced by diurnal rhythm, nor in the retest. (**B**) For the contextual fear conditioning, there is no difference in freezing time during the pre-shock session between active and inactive groups. Twenty-four hours after the footshock, both active and inactive groups show increased freezing time during the test session, but no significant difference was detected. Three months later, mice tested in active phase exhibited a higher percentage of freezing in the pre-shock session, but no difference in freezing time during the test session. (**C**) For active avoidance, mice tested in the active phase feature higher escape success rates compared to mice tested in the inactive phase. In the retest, no significant difference is detected. (**D**) The phase differences in expression of clock genes (*Per1*, *Per2*, and *Bmal1*) in the hippocampus at ZT4 (inactive group) and ZT16 (active group). Blue circles: male mice; Red circles: female mice.

Next, to better assess whether the circadian period affects other types of cognitive behavior, we conducted T-maze, contextual fear, and active avoidance tests. For T-maze alternation, the results show there were no differences in percentage of correct choices in the initial test (*p* = 0.79), nor the second test three months later (*p* = 0.8) (Fig. 4A). For contextual fear conditioning, there were no differences detected in freezing time during the pre-shock session (*p* = 0.07, *t*_(22)_ = 1.88), and there was no difference in freezing time detected in the test session 24 h after the shock (*p* = 0.15, *t*_(22)_ = 1.47) (Fig. 4B). Three months later, all mice were subjected to the footshock chamber again. Mice tested in the active phase exhibited an increase in freezing time during the pre-shock session (*p* = 0.007, *t*_(22)_ = 2.94), but no differences during the test session (*p* = 0.21, *t*_(22)_ = 1.27), suggesting mice tested in the active phase show better long-term memory of the footshock chamber (Fig. 4B). For active avoidance, mice tested in the active period exhibited higher escape success rate than those tested in the inactive phase (*p* = 0.0028, *t*_(22)_ = 3.36), suggesting that mice learn active avoidance better during the active phase. Three months later all the mice were subjected to active avoidance testing again, and the results show there was no significant difference (*p* = 0.25, *t*_(22)_ = 1.15) (Fig. 4C). Clock genes have been extensively studied and show circadian expression in the brain^11,12^. To further confirm physiological gene expression pattern of active and inactive periods in these mice, three days after the last behavioral test, we harvested hippocampal tissue for clock gene expression four hours after lights on/off. Our results show that hippocampal tissue harvested in the active period (Zeitgeber Time 16, ZT16) exhibit higher expression of *Per1* (*p* = 0.0001, *t*_(8)_ = 6.74) and *Per2* (*p* = 0.0013, *t*_(9)_ = 4.57), and lower expression of *Bmal1* (*p* = 0.0049, *t*_(9)_ = 3.69) compared to tissue harvested during the inactive phase (Zeitgeber Time 4, ZT4) (Fig. 4D). These results confirm the mice had differential gene expression between active and inactive phase.

### Sex differences in behaviors

In order to determine whether differences in sex could be contributing to our results, for each measure we compared a model in which phase predicted the behavior to a model that in addition contained sex as a predictor. We applied a Bonferroni 5% corrected threshold of 0.00156 to take into account the fact that we ran 32 tests (0.05/32 = 0.00156). Our figures show results for males and females where males are colored blue and females colored red. We found two behavioral results exceeded the 5% threshold; time spent in open arms (*p* = 0.00017) and travel distance (*p* = 0.0012) of elevated–O-maze at 20 weeks in our analysis. However, the small sample size and consequent low power means we cannot exclude the presence of a sex difference. Table 1 provides a summary of the sex difference analyses.

**Table 1:** Summary of sex difference analysis in each experiment.

Test	Time	p.sex	t.sex	Time	p.sex	t.sex
Rotarod (latency to fall)	8-week-old	0.88405	0.14762	20-week-old	0.05798	− 2.00536
Von Frey (left)		0.08414	1.81309		0.71014	0.37675
Von Frey (right)		0.10251	1.70728		0.00439	3.19124
Open field (travel distance)		0.29786	− 1.06750		0.16461	− 1.43998
Open field (time in center zone)		0.49743	0.69052		0.21310	− 1.28407
Elevated-O-maze (travel distance)		0.84313	− 0.20035		0.00127^†^	− 3.71962
Elevated-O-maze (time in open arms)		0.01552	− 2.63370		0.00017^†^	− 4.55817
Light–dark box (time in light box)		0.61658	0.50824		0.97763	− 0.02837
T-maze (correct choice)		0.31119	− 1.03775		0.52166	0.65170
Water maze acquisition (escape latency)		0.33181	0.99343		0.53841	− 0.62545
Water maze acquisition (travel distance)		0.06541	1.94401		0.62695	− 0.49325
Water maze memory (time in target zone)		0.23214	− 1.23043		0.90232	− 0.12422
Water maze memory (platform crosses)		0.31547	− 1.02837		0.16005	1.45646
Active avoidance (percentage of avoidance)		0.38343	− 0.89023		0.39436	0.86959
Contextual fear (pre-shock)		0.01358	− 2.69419		0.77097	0.29489
Contextual fear (test)		0.90799	0.11697		0.01173	− 2.76031

The table shows the behavioral tests used for each experiment. The t-statistic (t.sex) and the associated p-value (p.sex) are provided for the effect of sex (n = 12 in each sex) on the phenotype.

^†^*p* < 0.00156 (Bonferroni corrected threshold of 0.05/32 as the 5% significance threshold).

## Discussion

Mouse behavior is an important tool used to evaluate the effects of genes or experimental interventions in research. Mice are nocturnal animals, and few animal houses maintain mice under a reverse light system. In this study, we set out to investigate how marked differences are when behavioral tests are conducted during the different phases. At the beginning of the experiment, we assessed motor and sensory function. Our results show there is no significant difference in motor coordination, but mice tested in the active phase exhibit slightly greater cutaneous sensitivity. Indeed, previous studies have shown that the circadian rhythm influences the responses of mice in the von Frey test^13^.

Sleep is crucial for maintaining a normal emotional response^14^, and circadian period mutants have been shown to have altered anxiety in mice^15^. However, few studies explore anxiety across time of day^16^. Previous studies provide evidence that the expression of suprachiasmatic nucleus circadian oscillatory protein (SCOP) in the basolateral amygdala plays a key role in generating circadian rhythmicity in anxiety-like behavior^17^. Other studies show melatonin is involved in the anxiety response of diurnal and nocturnal species^18^. One previous study showed that mice tested at night or day performed differently in the light–dark box test, but displayed no significant difference detected in the open field^19^. In this study, we used three of the most commonly used tests for anxiety. In the initial test we did not observe remarkable differences between active and inactive groups. To further confirm these results, all mice were retested three months later. However, mice tested in active phase exhibited less travel distance in the elevated-O-maze, and spent less time in the light compartment in the light–dark box test. It is unclear if the difference at 20 weeks is because there is an age-dependent time of day effect or if there is a memory-dependent time of day effect. Whilst the initial exposure may have measured anxiety-like behavior, it may be the case that a subsequent exposure reflects a learned fear response in a mouse that remembers the original test.

The Morris water maze is one of the most popular tests to assess the performance of spatial learning and memory. Earlier studies in rats demonstrated that there is a modulating effect of diurnal rhythm on performance in the water maze^9^. However, our results show mice performed similarly in spatial learning and memory regardless of the phase in which they were tested. To better assess the effects of diurnal rhythm on learning and memory, we further investigated different types of learning and memory. For contextual fear conditioning, previous studies show that mice acquired conditioning faster in the day than in the night^20^. From our results, although mice tested in the active phase exhibit similar freezing time 24 h later, these mice showed higher freezing percentage in the pre-shock phase, 3 months later, indicating that mice tested in the active phase have more enduring memory for the footshock chamber. For the active avoidance test, we found mice tested in the active phase show higher escape success rate in the initial test. We did not detect differences in escape response in the retest experiment. One of the reasons for the lack of difference is that the mice show high avoidance responses in both groups, even though the retest was conducted three months after the initial test. Our data suggests that mice perform better in long-term contextual memory when trained in active phase, but not for spatial learning and memory.

In conclusion, we use two groups of mice to assess whether diurnal rhythm has a significant impact on sensorimotor, anxiety, learning and memory. First, we did not find a sex effect on most behaviors, but female mice featured lower anxiety in the elevated-O-maze test. Indeed, a large cohort study shows very limited evidence for sex differences^21,22^. Second, mice tested in active phase exhibited slightly more cutaneous sensitivity and better contextual memory and active avoidance escape response, but no difference in spatial learning memory and anxiety. However, behavioral tests, inevitably, will affect the biological rhythm, and the sleep of mice. The experimental results may be different if the mice tested in active phase were only exposed to red dim light. One thing to be noted, in this study we only tested mouse behaviors in different phases, without any intervention or genetic mutation involved. It is very likely that circadian rhythm will interact with the genes or treatment and potentially affect anxiety, learning or memory. Behavioral studies involving in genes manipulation or drug treatment, still need to take the diurnal rhythm into account.

## Methods

Experiments were performed on sex balanced cohorts of 8-week-old C57BL/6. Founders were ordered from National Laboratory Animal Center, Taiwan. After one generation of breeding, 4-week-old mice after weaning immediately were allocated in normal or reverse light animal room. Twenty-four mice were used in this experiment (n = 12 in each group; 6 males, 6 females). Mice were bred in AAALAC certified specific pathogen-free conditions. They were housed in a 12:12 h light dark cycle at a temperature of 22 °C and a humidity level of 60–70%. Animals had ad libitum access to food and water. All procedures were carried out in accordance with the local regulations and approved by Institutional Animal Care and Use Committee at Chang Gung University (Permit Number: CGU107-025).

### Behavioral testing

For behavioral tests, the movement of animals was recorded and tracked using a video recording system. Mice for conducting the behavioral analysis were randomized to a separate cage for testing, with bedding, food and water before being transferred back to the home cage. The behavioral data of anxiety and water maze was recorded and acquired automatically by Ethovision software.

#### Rotarod

Mice were held by the tail and placed on the rotarod (Ugo Basile), facing away from the direction of rotation. The rotarod moved at an initial speed of 4 rpm. After 10 s, the rod speed accelerated at a rate of 9 rpm per minute. Once acceleration had been triggered, the time taken for mice to fall was noted as previously described^23^.

#### Von Frey

Mice were placed individually in an elevated acrylic chamber equipped with a wire mesh floor, and a von Frey filament (BiosebLab) was applied from underneath pricking the hind paws. The strength of von Frey filament would continuously increase until paw withdrawal and the withdrawal threshold was recorded. Each mouse was pricked for five times both on the right hind paw and the left hind paw. The average withdrawal threshold of each hind paw was recorded.

#### Open field

Animals were allowed to freely move for 5 min in a circular-shaped area (radius = 60 cm) with a lamp being the only light source illuminating the center zone. The time spent in the center zone (radius = 40 cm) and the frequency of transition to that center zone were recorded by Ethovision software.

#### Elevated-O-maze

The maze was a circle (radius = 55 cm) elevated 60 cm above the floor. The closed arms featured a 15 cm wall. Mice were first placed in the closed arm and allowed to freely move for 5 min under dim light. The time spent in the open arms and the exploration distance were measured as described previously^24^.

#### Light–dark box

At the start of the test, mice were put in the covered dark box and allowed to move freely between dark and the coverless light boxes via a door. Mice were allowed to freely move in the chamber for 5 min under white light. The time spent in the light box and number of light box entries were recorded and analyzed by Ethovision software.

#### Water maze

Mice were put into the 130 cm-radius-water tank. The tank was filled with water at 22 degrees Celsius that was coloured with white acrylic turpentine. A circular 10 cm-radius-platform was submerged 1.5 cm below the water surface. Each mouse was trained to stay in the platform for 30 s per trial, three trials per day for 4 days. Mice were put into the tank at three different positions and faced to the wall of the tank. Seven days after the last training, we then performed the memory test with the platform removed. We recorded the time each mouse spent in the correct quadrant (target zone) and the number of times they crossed the phantom platform location.

#### T-maze

T-maze was performed as described previously^24^. Mice were put into the T-maze for 10 min one day before testing. Before the test, all guillotine doors were raised. The mice were placed in the start area and allowed to choose a goal arm. A mouse was confined to the chosen arm and start area by quietly sliding the other door down. After 30 s, the mouse was removed, and immediately place back into the maze to select an arm. Each mouse repeated the experiment three times. We recorded the percentage of correct times each mouse explored the novel arm.

#### Contextual fear and fear memory

Context-conditioned learning was assessed in a footshock chamber placed on a weight transducer (Panlab/Harvard apparatus) and analyzed with PACKWIN software. Mice were allowed to explore the chamber for 3 min. At the end of the trial, an electric shock with current intensity of 0.7 mA was given through the underlying conducting rods. Mice were placed into the same footshock chamber the next day for 3 min and the immobile time was recorded as described previously^24^.

#### Active avoidance

Mice were placed in a two-compartment shuttle box equipped with a speaker and a light bulb in each compartment (Med Associates, Inc). Subjects were given conditioned stimuli (5 s of light and 8 kHz, 85 dB tone) followed by an unconditioned electric footshock (0.3 mA) from the underlying conducting rods. Once the mice moved to the other compartment or the cut-off time (10 s) was up, the conditioned stimuli ceased. After a random inter-session interval (range 3–10 s), the next session started. Mice repeated 50 sessions in this test, according to protocol described previously^24^.

#### mRNA quantification

Hippocampal tissue was collected from all mice 4 h after lights on/off (ZT 4 for inactive group, ZT 16 for active group), then homogenized in Trizol, followed by Phenol–Chloroform extraction of total RNA. cDNA was then made using SuperScript III reverse transcriptase (Invitrogen). Experiments were performed in duplicate. Gene expression levels were then calculated with the ΔΔCt method and normalized against a *Gapdh* control, according to protocol described previously^25^. Below table are the primers sequences for PCR:

**Table:** 

*Per1* Forward	TTCAA GCTCT CAGGA CTCTG
*Per1* Reverse	GGCAG TTTCC TATTG GTTGG
*Per2* Forward	CAGGA GAAGC TGAAG CTGC
*Per2* Reverse	GGACT GTCTT CCTCA TATGG
*Bmal1* Forward	TGGAA GAAGT TGACT GCCTG GAAGG
*Bmal1* Reverse	GGCCC AAATT CCCAC ATCTG AAGTT AC

### Statistical analysis

The mean ± SEM was determined for each group. Statistical analysis was performed using Graphpad Prism software. Data were analyzed via an analysis of variance (ANOVA), *Mann*–*Whitney* U test (For T-maze), and *t*-test as appropriate. For sex differences in behaviors, we measured the significance of sex as the improvement in fit conferred by sex in a model where the phase (“inactive” vs “active”) predicted the behavioral measure. The significance of the fixed effect sex was assessed using an approximation to the sequential *F*-test based on the Wald test ^26^. We fitted models by REML (Restricted maximum likelihood), using the lmer function from the R package lme4 ^27^.

### Ethical approval

The study was carried out in compliance with the ARRIVE guidelines.

## Data Availability

The datasets generated and analysed during this study are available from the corresponding author on reasonable request.
